# Designing an intervention to improve cognitive evaluations in primary care

**DOI:** 10.1186/s43058-025-00693-1

**Published:** 2025-01-16

**Authors:** Kyra S. O’Brien, Kristin Harkins, MaryAnne Peifer, Melanie Kleid, Cameron Coykendall, Judy Shea, Jason Karlawish, Robert E. Burke

**Affiliations:** 1 Department of Neurology, University of Pennsylvania Perelman School of Medicine, 3400 Spruce Street, 3W Gates, Philadelphia, PA 19104 USA; 2 Leonard Davis Institute of Health Economics, University of Pennsylvania, Philadelphia, PA USA; 3 Department of Medicine, University of Pennsylvania Perelman School of Medicine, Philadelphia, PA USA; 4 Department of Family Medicine and Community Health, University of Pennsylvania Perelman School of Medicine, Philadelphia, PA USA; 5 Department of Medical Ethics and Health Policy, University of Pennsylvania Perelman School of Medicine, Philadelphia, PA USA

**Keywords:** Consolidated framework for implementation research, Modified Delphi method, Primary care, Cognitive impairment

## Abstract

**Background:**

Early diagnosis is crucial to the optimal management of patients with cognitive impairment due to Alzheimer’s disease (AD) or AD-related dementias. For some patients, early detection of cognitive impairment enables access to disease-modifying therapies. For all patients, it allows access to psychosocial supports. Patients typically first present their concerns about their cognition to a primary care provider, but in this setting, cognitive impairment is commonly underdiagnosed. There is also high variability in how cognitive evaluations are performed. We sought to understand barriers to and facilitators of cognitive evaluations in primary care, map barriers to implementation strategies, and gain consensus from stakeholders on possible strategies to improve dementia diagnosis in primary care.

**Methods:**

Semi-structured interviews conducted with primary care providers (PCPs). We used the Consolidated Framework for Implementation Research to inform our question guide and analysis, and incorporated chart-stimulated recall – using actual patients who had cognitive complaints who had presented to these providers – to understand clinicians’ medical decision-making processes. These data were used to map identified barriers and facilitators to targeted implementation strategies. Then, this candidate list of strategies was presented to an expert stakeholder panel including clinicians and clinical operations specialists. Through a modified Delphi process, the list was narrowed to select the most promising strategies to incorporate in an intervention to improve cognitive evaluations in primary care.

**Results:**

Twenty PCPs were interviewed and mentioned barriers included lack of expertise to perform or interpret an assessment, time pressures, lack of incentives, competing priorities, lack of decision-making supports, and limited access to dementia specialists. Facilitators included the presence of an informant or caregiver and having additional staff to conduct cognitive testing. Implementation mapping resulted in a list of 15 candidate strategies. Using the modified Delphi process, these were narrowed to six.

**Conclusions:**

We used a rigorous process to identify barriers to and facilitators of cognitive assessments in primary care, identify promising implementation strategies to address these barriers, and obtain the feedback of front-line users on these strategies. This holds substantial promise for improving cognitive assessments in primary care in future implementation trials.

**Supplementary Information:**

The online version contains supplementary material available at 10.1186/s43058-025-00693-1.

Contributions to the literature

- Research has shown that there are numerous barriers to performing evaluations for memory loss or other cognitive symptoms in the primary care setting; however, we lack effective interventions to address these barriers.
- We report a rigorous, structured approach to conducting a needs assessment, mapping implementation strategies to relevant barriers, and selecting strategies to include in an intervention to improve cognitive assessments in primary care.
- Our findings and methods fill gaps in the literature on detection of cognitive impairment in primary care, serving as a model for conducting dementia care improvement initiatives that can be implemented broadly.

## Background

Early detection of cognitive impairment can reduce the many harms of Alzheimer’s disease (AD) and AD-related dementias (ADRD) [1–3]. Once cognitive impairment has been detected, clinicians can provide care that decreases caregiver burden, improves patient quality of life, and reduces health care costs and utilization [1, 3–6]. For example, with early detection, patients are able to participate in shared decision-making and future care planning [7]. Precautions can be put in place to maintain and promote future physical, psychological, and financial wellbeing. In addition, novel anti-amyloid therapies for AD have been approved for clinical use in the United States and are being administered to patients in routine clinical practice [8, 9]. These medications may slow the progression of disabilities in persons with early disease – that is, mild cognitive impairment or mild stage dementia due to AD [10]. Thus, early detection of cognitive impairment is a necessity for access to potentially helpful AD treatments.

AD/ADRD diagnoses are often missed or delayed in primary care: prior work demonstrates 26–66% of dementia cases in this setting are unrecognized [11–14]. Mild cognitive impairment, the earliest stage of impairment in AD/ADRD, is quite rarely detected in primary care. One Medicare claims analysis of primary care patients estimated only 8% of expected cases of mild cognitive impairment were diagnosed [15]. Compared to geriatricians or neurologists, primary care providers (PCPs) less often follow guideline-recommended practices for cognitive evaluations, including use of standardized cognitive assessments, assessment of functional status, and use of brain imaging – despite being the most prevalent group to assess cognitive concerns [16, 17]. Additionally, studies have demonstrated variability amongst PCPs in aspects of dementia care, including diagnosis disclosure, patient and family counseling, and pharmacologic management [18, 19].

These findings – in concert with the rising prevalence of AD/ADRD – have led to systematic inquiry into why dementia is underdiagnosed in primary care [20–23]. For example, a survey conducted by the Alzheimer’s Association showed that a significant number of PCPs did not feel prepared to either diagnose or care for patients with AD. At least a third of PCP respondents refer patients to dementia specialists for diagnosis and care, despite there being too few specialists to meet this demand [24]. Patient, provider, and system level factors have been cited as barriers to diagnosis and care of patients with cognitive symptoms. At the patient level, these include demographic factors, disease awareness, and attitudes or stigma [22, 23]. At the provider level, clinical expertise, disease knowledge, and attitudes contribute [11, 21, 23, 25, 26]. At the system level, lack of administrative and community supports, lack of reimbursement, patient load, and time constraints impact care [21–23].

While these barriers might not be surprising, few studies have used methods informed by implementation science to assess these barriers and then use these data to guide intervention and implementation design. Even fewer have engaged front-line clinicians in the design of an implementation trial. We sought to use a structured process to: 1) conduct a pre-implementation assessment of barriers to and facilitators of cognitive evaluations in primary care practices, guided by the Consolidated Framework for Implementation Research (CFIR) [27], and 2) design an intervention to improve primary care cognitive evaluations via stakeholder consensus.

## Methods

### Overview

**Fig. 1:**
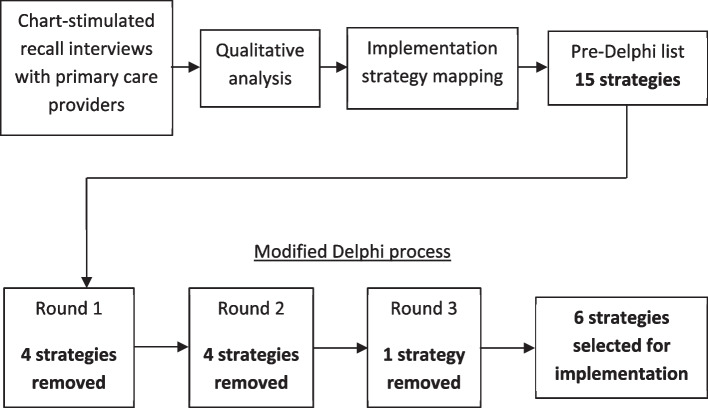
Project flow

This evaluation began with interviews of Penn Medicine primary care providers across Pennsylvania and New Jersey to learn about their cognitive evaluation practices and the factors that impeded or facilitated those evaluations. Interviews were structured using the CFIR, which allowed us to systematically analyze barriers and facilitators and map them to implementation strategies. Interviews also incorporated chart-stimulated recall, using clinicians’ own patient encounters to stimulate recall of their medical decision-making [28, 29]. Interviews were coded and analyzed, and a list of barriers and facilitators was created. We mapped the most salient barriers and facilitators to candidate strategies to address and leverage them, respectively. We then used a modified Delphi consensus process in which key stakeholders narrowed the list of candidate strategies to create an intervention to improve cognitive evaluations in primary care [30]. Figure 1 outlines this study flow. We adhered to the consolidated criteria for reporting qualitative research (COREQ – see Additional file 1) [31]. The interview study and modified Delphi protocols were reviewed by the University of Pennsylvania Institutional Review Board and deemed exempt. Participants reviewed a written description of the research and provided verbal consent to participate. Participant ID numbers were assigned as pseudonyms for anonymity.

### Sampling and recruitment

We recruited primary care providers (internal medicine and family medicine) with an active clinical practice whose patient population included individuals aged 50 or older and who had evaluated at least 4 patients for cognitive impairment within the prior year. These clinicians were identified through SlicerDicer, a data extraction tool in the Epic (Epic Systems Corporation, Verona, WI) electronic health record (EHR). We queried primary care visit encounters for a cognitive concern in the last year using International Classification of Diseases (ICD) 10 diagnosis codes for memory loss (R41.3), mild cognitive impairment (G31.84), or dementia (a diagnostic group inclusive of ICD-10 codes for all-cause dementia). We used this query to identify clinicians (included MD/DO physicians and advanced practice providers) who met the inclusion criteria. We prioritized recruitment from clinics with higher proportions of older adult patients and with larger medically underserved and/or racially and ethnically diverse patient populations. Email invitations to participate in the study were sent to the clinicians identified through the SlicerDicer query. A second recruitment email was sent after one week if no response was received. Individuals who did not respond after the second attempt were not contacted again. Demographic data were collected from participants via REDCap survey prior to their interviews [32, 33]. We aimed to interview between 20 and 30 clinicians, with the intent of establishing thematic saturation [34]. Clinicians received a $50 gift card for participation.

### Interviews

For each clinician who volunteered to participate, we used the SlicerDicer query described above to identify patient encounters for a cognitive concern in the last year. These charts were reviewed by the study team to select encounters for a new cognitive complaint (i.e., the patient complaint was not previously evaluated by any provider and/or the patient had not received a previous diagnosis of cognitive impairment by any provider). For each clinician, 2–4 encounters were selected by the study team using deviance sampling. Deviance sampling meant for each clinician we aimed to select at least one patient case with high adherence to recommendations for cognitive evaluations and at least one patient case with low adherence to these recommendations. Clinicians were asked to review the patient charts prior to the interview and rate their recall of the encounter from 0 (no recall) to 10 (perfect recall). We excluded charts for which recall was rated 0. The selected encounters were discussed during the Zoom interviews described below.

Semi-structured video conference interviews, lasting 30–40 min, were conducted between September 2022 and January 2023 by KO and MK via Zoom (Zoom Video Communications, Inc., San Jose, CA). KO is a female clinician-researcher (MD) with expertise in dementia care. KO works in the same health system as the PCPs invited for interview but does not work in the same clinic site as those invited. MK is a female research assistant with a bachelor’s degree and expertise in qualitative methods, with no prior relationship with participants. Both interviewers made field notes during the interviews. Prior to the interviews, participants were informed that the purpose of collecting these data was to inform the design of an intervention to support conduct of cognitive evaluations in primary care. The study interview guide (Additional file 2) was developed by the authors and piloted with one physician, after which minor revisions were made. In their interviews, participants were asked to describe barriers and facilitators to cognitive evaluations in the primary care setting, relying heavily on examples of their own patient encounters that they were prompted to recall. First, participants were asked to describe what happened in a recent encounter with a patient presenting with a memory complaint. Next, informed by CFIR domains, participants were asked to describe their perceptions of cognitive assessments (innovation domain) and how 1) elements of their office structure and health care system (inner and outer setting domains); 2) patient characteristics (individuals domain); 3) external influences (e.g., national mandates) (outer setting domain); and 4) personal experiences or perspectives (individuals domain) made cognitive evaluations easier or more difficult to perform in the primary care setting.

Finally, in a process known as chart-stimulated recall, participants were asked to discuss their medical decision-making for 2–4 of their own patient encounters selected through the EHR query. Eligible encounters were those that occurred within the previous year and in which an initial memory complaint was addressed. Clinicians were asked to discuss factors that made the cognitive evaluation easy or challenging to perform. Clinicians were able to access the charts during the interview for reference. As such, they were instructed to conduct the interviews in the privacy of their home or office without others present. Specific elements of a cognitive evaluation practices that were assessed were use of a validated cognitive assessment, obtaining informant history, and use of cognitive screening labs and brain imaging for patients found to have cognitive impairment. We assessed clinicians’ decision-making around performing these elements of the evaluation as well as the decision to refer to a cognitive specialist. Repeat interviews were not conducted.

### Qualitative analysis

We used deductive thematic analysis to look for patterns in the data and map data to predefined themes based on CFIR domains and constructs [27, 35]. We assembled a research team with diverse backgrounds, which helped us examine how our assumptions and motivations shaped study design, conduct, and data interpretation. We regularly incorporated such discussions into our meetings. Audio recordings were professionally transcribed. KO and MK checked for accuracy the transcripts of their interviews; they were not returned to participants for feedback, comment or correction. NVivo version R1/2020 (QSR International) was used to manage coding. Members of the research team (KO, CC, MK, KH) independently reviewed a subset of transcripts to identify themes, then met to discuss these themes and formalized them in a codebook—a taxonomy for categorizing qualitative data [36]. The codebook contained a mix of descriptive categories and interpretive categories.

Using the codebook, two authors (CC, MK) double coded a subset of six transcripts and met regularly during this process under the supervision of KO and KH to compare their coding, discuss discrepancies, and refine the codebook to rectify ambiguities, eliminate redundancy, and increase comprehensiveness. Having developed a refined codebook and agreement on its use, CC and MK then single coded the remaining transcripts.

After coding, the research team pulled out the data coded as barriers and facilitators and organized them according to CFIR domains and constructs. We incorporated four CFIR domains: 1) Innovation [37] – defined as the cognitive assessment, including obtaining a history, performing cognitive testing, and ordering diagnostic tests; 2) Inner Setting – defined as the primary care clinic; 3) Outer Setting – defined by two levels, the first being the Penn Medicine health system, and the second being the national policy climate; and 4) Individuals – defined as primary care clinicians. Within these domains, we identified the constructs that best mapped to our data, using the CFIR construct definitions described by Damschroder, et al. [27]. For example, within the Individuals domain, there are constructs of ‘Capability,’ ‘Opportunity,’ and ‘Motivation.’ We created summaries of our data for each domain. Finally, we created a list of the relevant constructs and the barriers and facilitators associated with them. For each, we identified the number of participants with whom that issue was discussed in order to identify which issues were most salient. We selected those barriers and facilitators mentioned by at least half of the participants to develop a list of candidate implementation strategies to support cognitive evaluations in primary care.

### Identification of candidate implementation strategies

A list of candidate strategies was developed using 3 methods: 1) selecting strategies from the Expert Recommendations for Implementing Change (ERIC) compilation via use of the CFIR-ERIC matching tool [38], which matches CFIR-based contextual barriers to implementation strategies best suited to address them based on expert consensus; 2) review of the literature for primary care interventions to improve cognitive evaluations and detection of cognitive impairment [39–47]; and 3) review of facilitators to performing cognitive evaluations that were reported by the clinicians who were interviewed.

The CFIR-ERIC matching tool, available at www.cfirguide.org, identifies the top seven ERIC strategies to address each CFIR construct selected. Level 1 strategies are defined as those endorsed by over 50% of an expert panel of implementation scientists and clinicians, and Level 2 strategies were those endorsed by 25%−49% of the expert panel. All Level 1 strategies (if any) and the top three Level 2 strategies for each construct were selected for the candidate strategy list. Strategies pertaining to the implementation process, such as creation of a stakeholder advisory panel, were not included in the list of candidate strategies reviewed in the modified Delphi process. Instead, these strategies have been incorporated into research planning and design. The CFIR-ERIC matching tool was developed using constructs from the first iteration of CFIR developed in 2009 [48], but the study described here used the updated CFIR version described in 2022 [27]. To use the CFIR-ERIC tool, we selected the original constructs that best matched the updated constructs based on their definitions.

### Modified Delphi consensus method

A modified Delphi method was used to pare down the list of candidate strategies to identify those most feasible, useful, and acceptable to key stakeholders. The Delphi process uses the input of expert stakeholders to address the research question and achieve consensus using surveys and participant communication [30]. The modification to the Delphi process allows for participants to provide their feedback via confidential, electronic surveys. Communication of ideas between participants was facilitated via email and distribution of de-identified free text survey responses to the stakeholder panel.

We used purposeful sampling to select stakeholders for the modified Delphi panel [49]. Clinician and clinical operations leaders with experience and influence relevant to the selected strategies were contacted for recruitment. Of the 14 individuals contacted, 11 agreed to participate. One left their position at Penn Medicine prior to the start of the consensus process. One individual did not respond after 2 contact attempts, and 2 individuals declined to participate. The final panel was comprised of ten stakeholders, all employees of Penn Medicine, including 2 primary care clinical operations specialists, 4 primary care clinicians (3 MD/DO, 1 advanced practice provider [APP]), and 4 specialists (2 neurologists, 2 geriatricians). The panel attended a brief 30-min introductory video conference call via Zoom, during which the research team explained the purpose of the panel and gave instructions for survey completion. All subsequent communications occurred via email.

Three rounds of surveys were used. Data for the first and second surveys were collected and managed using REDCap electronic data capture tools hosted at the University of Pennsylvania [32, 33]. The third survey was administered via Qualtrics (Qualtrics, Provo, UT). The first survey asked stakeholders to rate the candidate strategies’ feasibility, acceptability, and utility. Strategies were rated on a scale from 1 (low feasibility/acceptability/utility) to 5 (high feasibility/acceptability/utility) with an ‘Unsure’ option. Participants of different stakeholder types were only asked to provide ratings for the criteria relevant to that group, e.g., for a strategy that would be implemented by specialists, specialists were asked to rate its feasibility and acceptability, while primary care clinicians were asked to rate its utility and acceptability. Strategies receiving ratings of 4 or 5 for at least 75% of all responses were kept for the round 3 survey. Strategies receiving ratings of 4 or 5 for 50–74% of responses were kept for reconsideration in the round 2 survey. Strategies receiving ratings of 4 or 5 for less than 50% of responses were removed and not reconsidered. Participants were also asked to provide comments with justifications for their ratings in an open response field.

Prior to receiving the second survey, stakeholders received a summary of the responses as well as deidentified comments pertaining to the strategies to be re-rated. Panelists were invited to discuss any comments or concerns with the rest of the stakeholder group via email. The second survey presented the strategies to be reconsidered and asked participants to rate them using the same criteria used in the first survey. There was again an open response field for stakeholders to provide justifications for their ratings. Strategies receiving ratings of 4 or 5 for at least 50% of responses were kept for the round 3 survey.

Prior to the third survey, participants reviewed survey 2 results and deidentified comments. For this survey, participants selected the 3 strategies that they felt should be implemented out of groups of 5 strategies. Each survey contained all possible combinations of the strategies retained from rounds 1 and 2. This approach was taken so that stakeholders compared all strategies the same number of times and prioritized those they felt were most important. Any strategy receiving a median of 8 or more “keep” votes was selected for the final intervention, as this cutoff signified it was selected for implementation at least 50% of the time.

## Results

### Interview results

One hundred forty-six internal and family medicine clinicians across 15 primary care clinics were contacted for recruitment via email; 124 did not respond after two contact attempts and 3 declined to participate. We interviewed 20 primary care providers from 9 different clinics (5 urban, 4 suburban). Thirteen (65%) were MD/DO physicians and 7 (35%) were APPs. Six (30%) were family medicine providers and 14 (70%) were internal medicine providers. Fifteen (75%) participants were female. Half of participants had 10 or more years of experience in clinical practice. Twelve participants (60%) had worked in their current clinical practices for at least five years. Recruitment was stopped after 20 interviews, as the research team concluded that data saturation had been reached.

Coding identified barriers and facilitators associated with several CFIR constructs in the Innovation, Inner Setting, Outer Setting, and Individuals domains. Table 1 lists the relevant constructs and contains exemplary quotes within each CFIR domain assessed.

**Table 1:** Factors impacting cognitive evaluations in primary care

*CFIR Domain and Relevant Constructs*	*Exemplary quotations*
**Innovation:** **The Cognitive Assessment** *Adaptability* *Complexity*	**Adaptability:** “We serve a lot of patients who… come from areas where they didn’t graduate 12th grade… [the cognitive test] is probably not as accurate if you’re testing someone for memory loss and they have a highest education of maybe 6th or 7th grade.” (Participant 49) **Complexity:** “… could [the cognitive evaluation] be administered by a social worker or an MA if I just said… “Give them the paper,” and leave them in the room with it? But it’s not exactly like a PHQ-9 or something. It’s a harder test to explain.” (Participant 57)
**Inner Setting:** **The Primary Care Clinic** *Physical infrastructure* *Information technology infrastructure* *Team dynamics* *Culture* *Mission* *Relative priority* *Incentive systems* *Access to information*	**Incentive systems:** “It’s not like anyone is running around hitting us over the head saying, ‘You need to do these things.’ We’re rated on… mammograms and colonoscopies, and there’s that little thing on the dashboard for doing the memory evaluations. I’m completely mystified as to how they track that because my evaluations and what they say I’ve done don’t correlate whatsoever.” (Participant 36) **Relative priority, culture:** “[cognitive evaluations] is one of the many things we do in primary care, but I don’t feel [it’s] especially supported or emphasized… there’s more support around serious illness conversations, linkage to cancer and population health, mental health, but I don’t see that here… it’s just like a hole and everyone acknowledges it’s a hole.” (Participant 50)
**Outer Setting:** **Health System and Policy Climate** *Local attitudes* *Partnerships and connections* *Policies and laws* *Performance measurement pressure*	**Partnerships and connections:** “… it’s nice to have resources… locally. I often refer patients to the Office of the Aging, but that’s sometimes a hit or miss if they can get through.” (Participant 133) **Policies and laws:** “… the Medicare Wellness visit… it’s kind of like a preventive care visit… that’s nice that there’s that incentive to do the Medicare Wellness visit, but it doesn’t address… what if issues come up during that, and what do we do with those?” (Participant 12)
**Individuals:** **Primary Care Clinicians** *Capability* *Opportunity* *Motivation*	**Capability:** “It would be nice to have a lecture… to do something for primary care providers about evaluations, how to start that process of evaluating a patient and really retraining us.” (Participant 56) **Motivation:** “There’s the stigma of putting it on the chart IAQ worry about, and whether or not that will impact the patients’ relationship with other providers and how they communicate information to patients.” (Participant 113)

#### Innovation domain – the cognitive assessment

Within this domain, relevant constructs included the assessment’s adaptability and complexity. All participants noted that commonly used cognitive tests, such as the Mini Mental State Examination (MMSE) or Montreal Cognitive Assessment (MoCA) [50, 51], took too long to administer in the busy primary care setting. Many providers are allotted 15–20 min for a return patient visit, and conducting the cognitive test would not leave enough time for a history and exam. Additionally, most clinicians felt these tools were difficult to adapt for use in patient populations with language or literacy barriers. While most clinicians found these assessments were straightforward to administer, they reported difficulty interpreting the results of the evaluation and had low confidence in taking the appropriate next steps in the evaluation.

#### Inner setting domain – the primary care clinic

Within this domain, relevant constructs included the clinic and information technology infrastructure, connection and communication with other clinicians, clinic culture, tension for change, mission alignment, relative priority, incentive systems, available resources, and access to information. Primary care clinicians felt responsible for initiating cognitive evaluations, but the lack of time and resources to perform robust evaluations impeded this responsibility. Participants again noted the lack of time available to assess a patient’s cognition, due to short visit lengths and the wide array of health concerns a primary care provider is tasked with triaging. A few clinicians noted they would like assistance, either from medical students, medical assistants, or other available staff to conduct cognitive testing. Access to other resources within the clinic, such as social workers, interpreters, and dementia specialists, is also limited. Regarding social workers, clinicians felt that those working in primary care did not have adequate knowledge of dementia-related supports and thus could not address all patient needs. As for interpreters, live interpreters must be scheduled ahead of the visit, and this was not always being done. Finally, dementia specialists could be consulted by primary care providers, but there was a delay in receiving their input as the wait to see a specialist was several months long. Having these resources readily accessible in primary care would facilitate the cognitive evaluation and subsequent care. Clinicians also identified supports within the clinic space that made it easier to conduct cognitive evaluations, including easy access to cognitive testing forms and EHR tools to support decision-making and documentation.

#### Outer setting domain – the health system and policy climate

Within this domain, relevant constructs included local attitudes, partnerships and connections, laws and policies, and performance measurement pressure. Over half the clinicians noted there is little emphasis on cognitive care within the health system. While several initiatives and resources are dedicated to screening or management of other conditions, such as cancer and diabetes, the same is not true for cognitive assessments and dementia detection. Some clinicians indicated that either clear quality metrics or incentives would push some clinicians to conduct cognitive evaluations. Several clinicians noted that a lack of community partnerships made it difficult to connect patients to needed resources, some of which might facilitate further evaluation (e.g., transportation to appointments). Finally, the majority of clinicians discussed the impact of the Medicare Annual Wellness Visit, which requires screening for cognitive impairment [52]. Some clinicians felt this was a helpful way to keep cognitive health on their radar. Others did not feel this requirement was helpful, as there is not enough time to fully investigate cognitive concerns during the visit. There was also variability in how clinicians met the cognitive screening requirement for the Medicare Annual Wellness Visit. Some used only the patient interview to make this assessment, while others used the Mini-Cog, a validated cognitive screening tool consisting of three-word recall and clock draw [53].

#### Individuals domain – primary care clinicians

Within this domain, there were findings relevant to the capability, opportunity, and motivation domains. Most clinicians felt they lacked the expertise to perform a robust cognitive evaluation and desired more guidance on how to approach the evaluation, particularly for complex cases. Clinicians identified several specific topics for training, including how to tell whether cognitive symptoms are due to mood disorders rather than neurodegenerative conditions, when to obtain brain imaging, and how to handle patient distress during the evaluation. Clinicians endorsed that their longitudinal relationships with patients enabled them to recognize changes in their patients’ cognition. Some clinicians, often APPs, did not have this continuity in their patient panels and felt they had not developed a level of comfort with those patients that would be conducive to discussion of memory concerns. There was variability in clinicians’ motivation to conduct cognitive evaluations. Some felt a responsibility to perform these evaluations and felt they were on the front lines of dementia care, being the first to make recommendations for next steps and needed care. Others felt less motivated to pursue an evaluation as they perceived there to be limited treatment options for causes of dementia. Clinicians were also concerned about burdening patients with a diagnosis of cognitive impairment, believing it might cause fear or anxiety.

### Candidate implementation strategies

The Level 1 strategies derived from the CFIR-ERIC matching tool were: promote adaptability, conduct a local needs assessment, obtain patients/consumers and family feedback, organize clinician implementation team meetings, promote network weaving, identify and prepare champions, alter incentive structures, access new funding, conduct educational meetings, and develop and distribute educational materials. The Level 2 strategies derived from the CFIR-ERIC matching tool were: capture and share local knowledge, tailor strategies, develop a formal implementation blueprint, conduct ongoing training, involve executive boards, inform local opinion leaders, conduct local consensus discussions, change physical structure and equipment, create a learning collaborative, make training dynamic, and provide ongoing consultation. The results output for the CFIR-ERIC matching tool can be viewed in Additional file 3.

Several of these strategies were already incorporated into the research design, such as conducting a local needs assessment, organizing clinician implementation team meetings, promoting network weaving (e.g., conducting meetings that brought clinicians from different departments together with clinical operations specialists), and informing local opinion leaders. Strategies were tailored to address the specific issue of cognitive evaluations in primary care using a combination of interventions described in the literature, data from the semi-structured interviews described above, and discussions with local clinician and operations leaders. Table 2 contains the candidate strategies presented to the expert stakeholder panel in the first round of the modified Delphi process.

**Table 2:** Candidate strategies to improve cognitive evaluations in primary care

*CFIR domain*	*Strategy*
The cognitive assessment	Create a cognitive testing service within primary care, led by advanced practice providers, to administer the MMSE or MoCA and take relevant history. These visits can be in person or virtual
	Train clinic medical assistants to perform cognitive testing (MMSE or MoCA) and provide results to clinician
	Specialists create and disseminate to primary care providers an algorithm/decision tree for interpretation of cognitive testing
The primary care clinic and the health system	Have medical assistant place MoCA or MMSE worksheet in clinic room if the patient visit is for a cognitive evaluation
	Schedule cognitive evaluation visits for 30 min
	Send automated patient reminders to bring a family member with them if they are coming in for a cognitive evaluation
	Create and disseminate an electronic health record smart phrase containing list of relevant community resources for patients with cognitive impairment
	Have a social worker assist patients with scheduling follow up (with primary care OR a specialist) if they need additional testing for their cognitive evaluation
	Give primary care providers reports of the quality of their cognitive evaluations based on criteria such as use of a validated cognitive assessment tool and functional status assessment
	Introduce a new initiative, supported by leadership, to emphasize the importance of detecting cognitive impairment
	Partner with the Alzheimer’s Association Project ECHO to signify that cognitive evaluations are a priority in the health system
	Introduce interprofessional consults for access to dementia specialists’ expert opinion on clinical cases without needing patient to schedule an appointment to see a specialist
Primary care clinician knowledge and beliefs	Have primary care providers participate in live (in person or virtual) Continuing Medical Education (CME) training for cognitive evaluations
	Have primary care providers participate in virtual, asynchronous (not live) CME training for cognitive evaluations
	Provide annual lectures during regularly scheduled meetings (e.g., grand rounds) for primary care providers on updates in diagnosis and care of Alzheimer’s disease and related dementias, delivered by cognitive specialists and community partners

### Modified Delphi results

The 15-item list of candidate implementation strategies was shortened to six through the three rounds of the modified Delphi process. After the first round, 4 strategies were highly rated and kept on the list, 4 strategies received low ratings and were removed, and 7 strategies received either medium or polarized ratings on feasibility, acceptability and/or utility. In the second round, these 7 strategies were re-rated. Three of these strategies received majority support and were kept for the final round, and 4 of these strategies received low support and were removed.

In the final round, stakeholders rated and selected what they felt to be the top strategies from the remaining 7 strategies. Surveys contained 21 questions, each with one of 21 possible combinations of 5 of the 7 remaining strategies, i.e., each participant reviewed every possible combination. For each combination, participants were asked to select the top 3 strategies. In every survey, each strategy appeared 15 times. Strategies that were selected a median of 8 times or more were selected, and strategies selected less often were removed. The final list contained 6 strategies.

In the first two rounds of surveys, stakeholders were asked to provide an explanation for their ratings of the strategies. In general, strategies that were more time or labor intensive, especially for PCPs, received lower ratings. Some noted that without an incentive, it would be difficult to ensure PCPs attended educational lectures on cognitive assessments. There was also concern that asking for more time from PCPs would lead to greater burnout. Strategies that involved bringing more support into the primary care clinic, such as from dementia specialists and social workers, received higher ratings.

### Selected intervention components

Table 3 shows the final six strategies the stakeholder panel recommended for implementation through the modified Delphi process and how often those strategies were selected in the final survey. These strategies were 1) An APP-run cognitive testing service, 2) A decision algorithm for interpreting cognitive testing, 3) An EHR-accessible community resource list, 4) Assistance with scheduling cognitive follow up visits, 5) Interprofessional consults with dementia specialists, and 6) Annual AD/ADRD education for primary care. The strategy with the highest selection frequency was use of interprofessional consultation for primary care providers to access dementia specialists’ expert opinion on clinical cases (selected a median of 15 times, interquartile range [IQR] 1.5). Creation of an APP-led cognitive testing service had the greatest variability in selection (selected a median of 8.5 times, IQR 13.5). In general, the clinical operations specialists prioritized the APP-led cognitive testing service and provider education, while the specialist and primary care clinicians prioritized the clinician decision-making supports and patient resources and supports.

**Table 3:** Strategies selected for implementation through the modified Delphi method

*Strategy*	***Selection frequency – median (IQR)***
Create a cognitive testing service within primary care, led by advanced practice providers, to administer the MMSE or MoCA and take relevant history. These visits can be in person or virtual	8.5 (13.5)
Specialists create and disseminate to primary care providers an algorithm/decision tree for interpretation of cognitive testing	12 (6)
Create and disseminate an electronic health record smart phrase containing list of relevant community resources for patients with cognitive impairment	10.5 (10.25)
Have a social worker assist patients with scheduling follow up (with primary care OR a specialist) if they need additional testing for their cognitive evaluation	12.5 (9)
Introduce interprofessional consults for access to dementia specialists’ expert opinion on clinical cases without needing patient to schedule an appointment to see a specialist	15 (1.75)
Provide annual lectures during regularly scheduled meetings (e.g., grand rounds) for primary care providers on updates in diagnosis and care of Alzheimer’s disease and related dementias, delivered by cognitive specialists and community partners	9 (11.5)

## Discussion

Using semi-structured interviews and a modified Delphi consensus method, we identified and selected strategies to improve cognitive evaluations, tailored to the primary care setting. These strategies included support for clinicians for conducting and interpreting cognitive testing, including ready access to dementia experts, and patient supports for scheduling appointments for cognitive care and accessing community resources. Semi-structured interviews employing chart-stimulated recall provided rich data on determinants of the quality and extent of these evaluations. These determinants were mapped to candidate implementation strategies. The list of strategies was narrowed and then prioritized via consensus among relevant stakeholders using a modified Delphi method.

The barriers and facilitators elicited in the primary care clinician interviews align with what is described in the literature [11, 23]. We learned how PCPs have difficulty conducting cognitive testing when faced with short visit lengths, competing patient priorities, and lack of staff support. Additionally, many PCPs felt they lacked the necessary training to complete a cognitive assessment. Many of the PCPs we interviewed noted a tension between feeling responsible for diagnosing and managing cognitive impairment and not having the ability to do so because of practice and health system constraints. This is consistent with findings in another qualitative study examining primary care providers’ views on their role in dementia diagnosis and care, where they reported several systemic barriers to providing comprehensive assessment and management, such as lack of time, staff support and resources, and reimbursement [54].

Though the challenges of detecting cognitive impairment and dementia in primary care are well-documented, there have been few successful interventions to mitigate those barriers. Different strategies have been proposed or tested, such as clinical decision-making supports embedded into the EHR and educational programs to train primary care providers in dementia diagnosis and care [43, 45]. Evidence is mixed for some of these interventions [44]. One study demonstrated that educational interventions and decision support systems improved PCP detection of dementia, but did not improve adherence to clinical practice guidelines for the diagnosis and management of dementia in primary care [42]. Others have reported that educational interventions improved PCP confidence in the diagnosis and management of dementia and changed clinical practice, but these outcomes were measured by PCP self-report, without a direct assessment of effects on clinical practice by researchers [43, 46, 47].

Notably, these studies did not describe a pre-implementation assessment of the practice context, nor did they employ a framework to guide implementation efforts. As discussed above, previous interventions to improve dementia detection in primary care have focused mainly on clinician education and decision-making, which does not address health system or patient-related barriers. Our structured approach, using the CFIR and stakeholder engagement, allowed us to select strategies that reduce the time and work demanded of PCPs and support patients’ access to care. As we proceed with testing and refining this intervention, we will continue to use these frameworks to design, conduct, and assess its implementation. This work may also build evidence to support the broader use of implementation science methods in dementia care improvement initiatives.

This study has strengths and limitations. The use of chart-stimulated recall was beneficial as it allowed us to understand real instances of medical decision-making rather than hypothetical situations. As for limitations, interviews were conducted with a limited sample of primary care clinicians across a single health system, and the participation rate was low among those contacted for recruitment. These limitations impact the generalizability of the findings, and there may be selection bias. However, as our intention was to learn about context-specific barriers and facilitators, the findings will support design of an intervention tailored to our health system. Further, the methods utilized to conduct this evaluation are generalizable to other contexts and can be used to design new interventions or adapt core components of an existing intervention. Future studies should aim to understand variations among cognitive evaluation practices in urban, suburban, and rural communities to better understand how intervention components may be tailored to those settings. Another limitation is use of the CFIR-ERIC tool. While it provides a structured tool for implementation strategy mapping, there is not yet evidence that use of the tool produces effective strategies.

A strength of our study was use of the modified Delphi method, which allowed us to narrow our intervention to focus on strategies considered by diverse stakeholders to have higher feasibility, acceptability, and utility. We chose to conduct the Delphi process via electronic communication to accommodate stakeholders’ busy schedules. This removed the opportunity for face-to-face discussions which could have impacted stakeholders’ ratings of the strategies. A limitation of our Delphi process was the lack of patient and family representatives on the Delphi panel, as strategies targeted to clinician workflow would also impact the patient experience. However, we felt it would be difficult for patients and families to weigh in on the feasibility, acceptability, and utility of strategies that were largely targeted towards clinician workflows. We will include patient and family stakeholders in the next phases of implementation planning and execution.

## Conclusions

Primary care providers are expected to play a critical role in early detection of cognitive impairment, but there are barriers that must be addressed to achieve this. Through a structured evaluation employing an implementation science framework, we have created a multicomponent intervention comprised of six strategies selected by expert stakeholders to aid primary care providers with the cognitive evaluation. Implementation of these strategies will be planned, executed, and evaluated by multidisciplinary stakeholder teams, with the goal of refining the intervention and testing it at a larger scale.

## Supplementary Information

Additional file 1. COREQ Checklist; this file contains the COREQ reporting guidelines checklist for the interview portion of this study.

Additional file 2. Study Interview Guide; this file contains the interview guide used for the qualitative portion of this study.

Additional file 3. CFIR-ERIC Mapping; this file contains the output of the CFIR-ERIC matching tool for this study.

## Data Availability

The datasets used and/or analyzed during the current study are available from the corresponding author on reasonable request.

## Abbreviations

AD: Alzheimer’s disease
ADRD: Alzheimer’s disease-related dementias
APP: Advanced practice provider
CFIR: Consolidated Framework for Implementation Research
COREQ: Consolidated criteria for reporting qualitative research
EHR: Electronic health record
ERIC: Expert Recommendations for Implementing Change
IQR: Interquartile range
MMSE: Mini Mental State Examination
MoCA: Montreal Cognitive Assessment
PCP: Primary care provider
