# Supplementary material for: Designing an intervention to improve cognitive evaluations in primary care
Source: Implement Sci Commun. 2025 Jan 16;6:9. doi: 10.1186/s43058-025-00693-1 (PMC11740457; doi:10.1186/s43058-025-00693-1)
Supplement: Supplementary file 2 — Additional file 2. Study Interview Guide; this file contains the interview guide used for the qualitative portion of this study. [file 43058_2025_693_MOESM2_ESM.docx]

Thank you for taking the time to meet with me today. The purpose of this study is to understand how patients are evaluated when they present to their primary care provider with a cognitive complaint, such as memory loss. First, I’d like to get a general sense of how you approach this kind of patient and some of the things that can make it harder or easier to assess a patient for memory loss. Then we’ll discuss some of the patient encounters we asked you to review. Do you have any questions before we get started?

1. I’d like you to think about a recent patient you evaluated for memory loss. Tell me about how you approached to the evaluation of their memory complaint.

Probes:

a. What information do you try to obtain?

b. Do you typically dedicate an entire visit to the evaluation or might you also be evaluating other medical problems at the same time?

c. What lab testing, if any, might you obtain (ie. blood work, imaging)?

1. What usually leads to you performing an assessment of a patient’s memory – for example, is it triggered by a patient complaint, family complaint, or do you notice changes?

Probes:

a. Does your approach differ depending on who brings up the issue initially?

I imagine that there can be different types of challenges to evaluating a patient for memory loss. For example, some may be related to the cognitive evaluation itself while others might be related to office structure or the healthcare system.

1. Let’s start with talking about the cognitive evaluation itself (for example, taking the history, doing a cognitive assessment and ordering testing). What aspects of the cognitive evaluation can make it more difficult to perform?
2. Ok, now what aspects of the cognitive evaluation can make it easier to perform?

*Probes for 3 and 4:*

How do/does _____ impact your ability to do a cognitive evaluation?

a. Evidence behind recommended practices

b. Complexity

c. Adaptability for primary care

1. What characteristics of a patient can make it more difficult to evaluate cognitive symptoms?
2. What patient characteristics can make it easier to perform a cognitive evaluation?

*Probes for 5 and 6:*

How do/does _____ impact your ability to do a cognitive evaluation?

a. Comorbidities/other needs

b. Access (to resources, support)

c. Health literacy

d. Lack of caregiver

1. What features of your practice and/or the health care system make it more difficult for you to evaluate a patient’s cognitive symptoms?
2. What features of your practice and/or health care system make it easier for you to do a cognitive evaluation?

*Probes for 7 and 8:*

How do/does _____ impact your ability to do a cognitive evaluation?

a. Clinic/health system priority of memory care

b. Organizational incentives

c. Resources (ie. training, time, space, money)

d. Leadership support

1. Tell me about any influences that are external to your practice and health care system that make it more difficult to evaluate a patient’s cognitive symptoms?
2. What influences external to your practice and health care system make it easier to do a cognitive evaluation?

*Probes for 9 and 10:*

How do _____ impact your ability to do a cognitive evaluation?

a. National mandates

b. Best practice guidelines

1. Tell me about any personal perspectives on or experiences with cognitive symptom evaluations that might make it more challenging for you to do a cognitive evaluation.
2. What personal perspectives on or experiences with cognitive symptom evaluations make it easier to perform a cognitive evaluation?

*Probes for 11 and 12:*

a. What do you think is the value of detecting cognitive impairment?

b. What do you think is the role of primary care in detecting cognitive impairment?

Is there anything else you would like to mention before we discuss the patient encounters?

Ok, for each case I will ask a series of questions to better understand how patients with cognitive concerns are assessed in primary care. The goal of this is to help identify ways to improve the process that make sense for primary care providers. Using real-life examples makes it much more tangible. Our goal is not to pass judgment in any way, but please let me know if you feel uncomfortable with the conversation at any point. You are welcome to open the patient’s chart if you need to review anything while we are talking – I can give you the patient’s MRN if you would like to look them up.

[Indicate which case we are reviewing]

1. How well did you know this patient before this visit; had you been seeing them for a while or only recently met them?

2. Did they come in primarily for a memory concern or for a different or multiple reasons?

3. At this visit:

1. What history did you take and from who (e.g the patient, and informant, or both)?
2. Did you assess the patient’s functional status (ie. discuss activities of daily living)? Tell me about that decision.
3. Use a cognitive assessment tool such as the MMSE, MoCA or SLUMS? Tell me about that decision.
4. Was any diagnostic testing (blood work and/or imaging) ordered?

i. If yes, what tests were ordered and why?

ii. If no labs and/or head imaging ordered, why not?

1. Was the patient referred to another provider for evaluation? Tell me about that decision.

4. What challenges did you face in evaluating this patient’s cognitive symptoms?

5. What things helped you evaluate this patient’s cognitive symptoms?

Thank you so much for providing these valuable insights. Is there anything else you would like to mention before we wrap up?
